# Disparities in being able to donate human milk impacts upon maternal wellbeing: Lessons for scaling up milk bank service provision

**DOI:** 10.1111/mcn.13699

**Published:** 2024-07-10

**Authors:** Amy Brown, Catrin Griffiths, Sara Jones, Gillian Weaver, Natalie Shenker

**Affiliations:** ^1^ Centre for Lactation, Infant Feeding and Translation (LIFT) Swansea University Swansea UK; ^2^ Faculty Medicine, Health and Life Sciences Swansea University Swansea UK; ^3^ The Human Milk Foundation, Gossams End Berkhamsted UK; ^4^ Department of Surgery and Cancer Imperial College London London UK

**Keywords:** breastfeeding, donation, donor human milk, health service delivery, lactation after loss, mental health, milk bank

## Abstract

Receiving donor human milk for a baby can have a protective effect upon parental wellbeing. A growing body of research also finds that being able to donate milk to a milk bank, particularly after infant loss, can also boost maternal wellbeing through feelings of altruism and purpose. However, most studies are qualitative, with small sample sizes outside the United Kingdom, and often do not include the experiences of those who have been unable to donate. Our aim was therefore to examine the impact of being able to donate milk, as well as the impact of not being able to do so, using a survey containing open and closed questions in a large UK sample. Overall, 1149 women completed the survey, 417 (36.3%) who donated their milk and 732 (63.7%) who did not. Most women who donated found it had a positive impact upon their wellbeing, feeling proud, useful and that they had achieved something important. Conversely, those unable to donate often felt rejected, frustrated, and excluded, especially if they received no response or felt that restrictions were unfair. Thematic analysis found that being able to donate could help women heal from experiences such as birth trauma, difficult breastfeeding experiences, neonatal unit stays, and infant loss; however, being unable to donate could exacerbate negative emotions arising from similar experiences. A minority of women who donated experienced raised anxiety over following guidelines. These findings further extend the impacts of milk banking services beyond infant health and development and support expanded service delivery.

## Key messages

- Mothers who could donate felt proud, useful and a sense of achievement but those who could not often felt rejected, frustrated and excluded.
- Donating could help to heal, whereas inability to donate could exacerbate challenging experiences such as infant loss, birth trauma, breastfeeding difficulties, and perinatal anxiety and depression.
- Some women were unable to donate for unavoidable reasons such as medication or lifestyle factors. However, others were prevented for potentially modifiable reasons including distance from a milk bank, receiving guidance too late or not receiving a response, highlighting priorities for service expansion and a greater awareness of milk donation antenatally.

## Graphical abstract

Our findings show that being able to donate milk was associated with feelings of pride, achievement and altruism. Donation could help women to heal after birth trauma, difficult breastfeeding experiences, neonatal unit stays and infant loss (pictured). However, being unable to donate could exacerbate feelings of grief, loss and guilt.

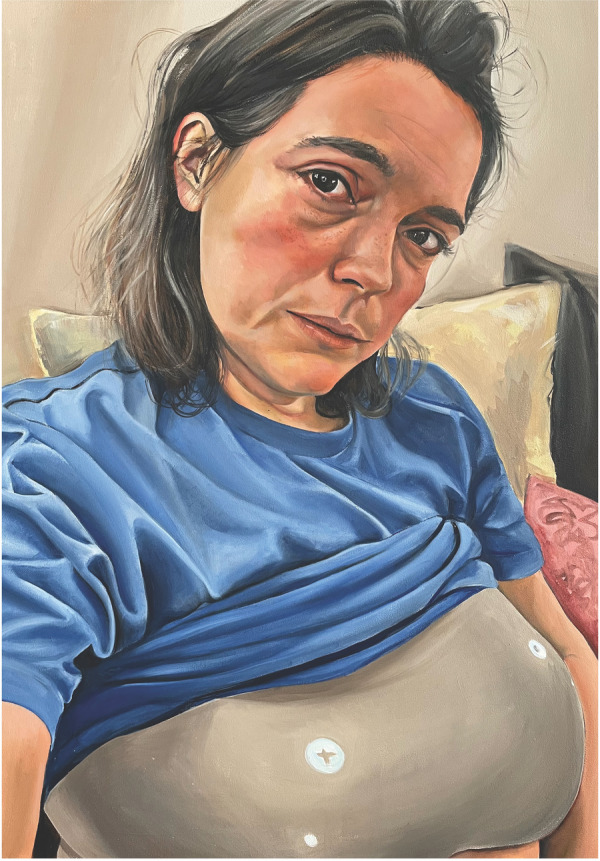

## 1 INTRODUCTION

It is well established that breastfeeding and human milk protect maternal and infant health (Victora et al., 2016). When babies cannot receive their own mother's milk, donor human milk (DHM) may protect premature infant health and development, reducing the risk of necrotizing enterocolitis and other complications such as bronchopulmonary dysplasia (Quigley et al., 2019; Villamor‐Martínez et al., 2018). It is most important to ensure that mothers receive the lactation support needed to establish breastfeeding and maximize their own milk supply. However, where there is an interim shortfall in maternal milk for a premature infant on the neonatal unit, or when breastfeeding is not possible at all for reasons such as maternal cancer, research is building that shows that receiving DHM can help to protect parental mental health and wellbeing through both reduced anxiety for infant health and the feeling of being supported and respected (Brown & Shenker, 2022; Brown et al., 2024; Cassidy & Dykes, 2019; Kair & Flaherman, 2017). Mothers can also feel that being able to give their baby human milk helps to heal the grief experienced when unable to breastfeed, or alternatively supports them to increase their own milk supply if possible (Brown & Shenker, 2022; Brown et al., 2024). Recent guidelines published by the British Association of Perinatal Medicine therefore emphasized that parental wishes regarding feeding should be considered and recognize that DHM can help to support maternal wellbeing when exclusive breastfeeding is not possible (BAPM, 2023).

The importance to women of being able to donate their milk is increasingly recognized (Doshmangir et al., 2019) and milk banks have an ethical obligation to provide support to donors throughout their donation period (Hartmann, 2017). One survey of 50 donors in the United States highlighted the positive impacts of donation, driven by reasons such as having more milk than their baby needs and therefore feeling that their milk would not be wasted, their own baby having received DHM and wanting to ‘pay back’, and wanting to help other families in need (Wambach et al., 2019). Another survey of 72 donors in Sweden also found that women enjoyed the feelings of altruism, appreciation and feeling that their milk had gone to good use (Olsson et al., 2021). Qualitative studies exploring these experiences in more depth describe feelings of pride, joy, hope for the future and how many mothers felt like ‘a better person’ after donating (Candelaria et al., 2018; Miranda et al., 2016). Donation can be particularly meaningful for mothers after baby loss, helping them to feel meaning in their experience, overcome grief and feelings of their body failing, feel connected to and honour their baby and feel that their baby lives on and is helping others (Cassidy & Dykes, 2019; Cole et al., 2018; Fernández‐Medina et al., 2022; Oreg, 2020; Waldby et al., 2023; Ward et al., 2023; Welborn, 2012).

However, challenges sometimes arise from milk donation that can feel difficult or distressing to mothers. For example, issues such as ensuring that their own baby has enough milk, disliking blood tests, the time it takes to express, potential discomfort or pain from expressing, parting with expressed milk, practicalities such as keeping track of expiration dates and storage guidelines and transporting milk can feel difficult. Some felt that their efforts were not appreciated or that they did not get the support needed (Olsson et al., 2021; Wambach et al., 2019). Some women in these circumstances go on to share their milk with others in their family or community and appear to experience similar feelings of satisfaction in supporting others (Gribble, 2013, 2014).

Sometimes mothers are unable to donate their milk even when they wish to. Issues such as medication use, transport/location challenges and not being able to meet minimum quantities can prevent donation (Biggs, 2021; Gribble, 2013). However, there is a dearth of research exploring the emotional impact of milk donation. In one study, mothers who were unable to donate or continue donating milk due to health, logistical or infant age limits felt frustrated and rejected (Hyde et al., 2023). This is an important area to explore, because opportunity to donate milk if desired is not currently equitable. In the United Kingdom, logistical transport issues can arise if a mother does not live within a certain radius of a milk bank. National Institute for Health and Care Excellence (NICE) clinical guidelines provide recommendations regarding human milk donation based on health factors such as infection status (HIV, Hepatitis B and C, Syphilis) and lifestyle factors such as alcohol intake and smoking (NICE, 2010). However, other recruitment criteria vary markedly between milk banks, leading to inconsistencies around specific guidelines between milk banks such as for age of infant at first donation. Women who currently are unable to donate their milk to a milk bank may choose to provide milk to a commercial company operating in the United Kingdom, in exchange for financial compensation. The operating practices of commercial human milk practices have only recently been described in studies that highlight a range of potential harms to providers and their infants (Newman & Nahman, 2022; Shenker et al., 2024), and commercial products have only recently started to be marketed to National Health Service hospitals in the United Kingdom (Steele & Cooke, 2024), but the experiences of women who contact them remain unexplored in comparison to altruistic milk donation.

Milk donation has therefore been shown to be associated with aspects of maternal wellbeing, but studies tend to be qualitative, do not directly focus on wellbeing and do not compare the experiences of mothers who are able or unable to donate. There is also little research considering milk donation and wellbeing within a UK setting, separate to the act of milk sharing or trading in the community, or the provision of milk to a commercial company. The aim of the current study was therefore to conduct a mixed methods exploration of the emotional impacts of milk donation amongst a larger sample of mothers in the United Kingdom.

## 2 METHODS

### 2.1 Participants

Participation was open to any woman in the United Kingdom, who had wanted to donate her breast milk to a milk bank, whether she was able to do so or not. We made the decision not to restrict eligibility by donors’ age or the time elapsed since wanting to donate milk, to allow insight into the impact of time on donation or nondonation experiences. We recognize the potential recall bias of this decision and apply caution to those recalling experiences from a longer duration. Further inclusion criteria were aged 16+ years, able to complete the survey in the English language and able to give informed consent.

Data were collected between May 2022 and March 2023. The study was first conducted in Wales (May–September 2022) specifically due to changes in donation availability in the region at the time. Participation was then opened up across the United Kingdom (November 2022–March 2023). This meant that a higher proportion of participants (19.8%) were from Wales compared to the proportion of the UK population in Wales (4.6%). However, Wales is the only country in the United Kingdom not to have its own milk bank. In 2021, a milk bank hub was set up in South Wales allowing increased milk donation ability in the region. Before this period, it was difficult for those living in south, west and mid Wales to donate. Over sampling in this area enabled us to explore the experiences of the subgroup who were not able to donate due to logistical reasons but should not necessarily be considered representative of population‐level experiences.

Full ethical permission for the study was gained from Swansea University School of Health and Social Care Research Ethics Committee. Participants gave informed consent and all aspects of the study were carried out in line with the declaration of Helsinki. All participants gave informed consent before completing the survey.

### 2.2 Measures

Participants completed an online survey hosted via Qualtrics (for the full set of questions please refer to Supporting Information S1: Appendix 1). The survey consisted of a series of tick box, Likert scale and open‐ended questions:

- Demographic details (age, education, location)
- Reasons for wanting to donate milk
- Donation details (whether donated, where and when, reasons for not being able to donate)
- Perceived impact of being able to donate or not
- Specific perceived impacts upon mental health

Participants also completed a series of open‐ended questions exploring their motivations and experiences of being able to donate milk or not:

- All participants: Were there any other reasons why you wanted to donate your milk?
- Able to donate: Can you describe any other impacts of how being able to donate milk made you feel? What were the most positive outcomes of being able to donate milk for you? What did you find challenging or stressful? Do you feel that the experience of donating had any significant impact upon your mental health?
- Unable to donate: Can you describe any other impacts of how being unable to donate milk made you feel? Do you feel that the experience of not being able to donate had any significant impact upon your mental health?

### 2.3 Procedure

Study adverts were placed on social media by the team with details of the study. Posts were shared on the academic/organizational pages of the research team including Instagram, Facebook pages and Twitter with encouragement for interested viewers and organizations to share further. The adverts were shared at least 200 times over the data collection period (privacy settings prevent a specific number being calculated) with metrics suggesting a post reach of at least 250,000 accounts.

If potential participants were interested in finding out more, they clicked on a link to take them to the participant information sheet and consent form. If inclusion criteria were met and consent given, the full survey loaded. Once completed, a debrief statement was given, explaining the study, thanking them for participation and giving them contact details for support organizations, if needed.

### 2.4 Data analysis

Data were analysed using SPSS version 29. Descriptive statistics were used to compute score frequencies and multivariate analysis of variance were used to examine differences between groups in the quantitative data. Qualitative data from the open‐ended questions was analysed using surface‐level thematic analysis (Braun & Clarke, 2006). Author CG immersed themselves in the data, reading through responses from each participant and across questions for all participants. Next, responses were read and re‐read to identify smaller themes. These smaller themes were then grouped into larger subthemes (Braun & Clarke, 2006). To enhance trustworthiness of the data (Lincoln & Guba, 1986), author AB then reviewed proposed themes and subthemes. Where disagreement occurred, themes were discussed until agreed.

### 2.5 Ethics statement

Ethical approval was granted by the Research Ethics Committee at the College of Human and Health Sciences, Swansea University

## 3 RESULTS

This section describes the participants and reports reasons for wanting to donate milk, donation rates, the quantitative and qualitative results of mothers able to donate (eight themes), and the quantitative and qualitative results of mothers unable to donate, (six themes).

Overall, 1149 mothers completed the questionnaire. Mean age was 34.54 (SD 5.87) with a range from 20 to 74 years. The majority of the sample (98.4%) were aged 50 years or younger. Further demographic details are found in Table 1.

**Table 1:** Participant demographic background (*n* = 1149).

Category	Subcategory	*N*	%
Age (years)	18–24	39	3.4
	25–29	143	12.4
	30–34	416	36.2
	35–39	368	32.0
	40–44	135	11.7
	45+	48	4.1
Education	No formal qualifications	1	0.1
	GCSE or equivalent	33	2.9
	A level or equivalent	142	12.4
	Degree or equivalent	490	42.6
	Postgraduate qualification or equivalent	481	41.9
	Prefer not to say or missing	2	0.2
Ethnicity	Asian or Asian British: Bangladeshi	2	0.2
	Asian or Asian British: Chinese	5	0.4
	Asian or Asian British: Indian	10	0.9
	Asian or Asian British: Pakistani	3	0.3
	Any other Asian background	3	0.3
	Black or Black British	11	0.9
	Mixed or Multiple	35	3.0
	White British or Irish	998	86.8
	White (other)	69	6.0
	Gypsy or Irish Traveller	2	0.2
	Any other group	2	0.2
	Prefer not to say	8	0.7
Number of children	First‐time mother	492	42.8
	Second time or more mother	657	57.2
Country	England	775	67.4
	Northern Ireland	36	3.1
	Scotland	110	9.6
	Wales	228	19.8

### 3.1 Reasons for wanting to donate milk

All participants were asked what led them to want to donate their milk. Participants could select multiple options with a further open‐ended box to describe additional reasons (Table 2). Almost all women were motivated by the idea of helping other families with around half having excess milk that they wanted to be used. In the open‐ended text box, participants gave further reasons for wanting to donate. Additional identified reasons are shown in Table 3, highlighting the diversity of both motivations and potential positive wellbeing impacts of donation.

**Table 2:** Reasons for wanting to donate (showing number of participants who selected each reason, *n* = 1149).

	*N*	%
I liked the idea of helping other families	1038	90.3
I had much more milk than my baby needed	553	48.1
I found expressing milk easy	507	44.1
I read an article/heard a news item about donor milk	429	37.2
I had expressed milk, but my baby didn't need it	400	34.8
I had expressed milk, but my baby wouldn't drink it	255	22.2
My baby was in neonatal care	193	16.8
I had donated milk in the past	163	14.2
Friends/family had donated	124	10.8
My baby received donor milk	111	9.7
A health professional told me about milk donation	98	8.5
My baby died	19	1.7

**Table 3:** Additional reasons for wanting to donate milk.

Category	Reason	Example
Health reasons	Infant allergies	‘Expressed milk stash for 4 months but found out baby had a dairy allergy and didn't want it to go to waste’
	Own allergies	‘I have many food allergies myself and wanted to donate to mothers who wanted to give their baby breastmilk but struggled to exclude from their diet foods I'm allergic to (and therefore do not consume)’
Mental health and wellbeing	Helping heal a difficult breastfeeding journey	‘After ending up mostly expressing due to my baby developing bottle preference when I returned to work, and having had a very difficult breastfeeding journey from the start, donating helps me to feel more of a sense of success ‐ a positive to come out of the fact that my breastfeeding journey doesn't look how I wanted or planned’.
	Own wellbeing	‘It meant I could eat more chocolate. It made me feel useful when suddenly I was on maternity leave and wasn't being useful at work’
	Difficult birth	‘I had a bad birth experience and it made me feel empowered’.
	Passion about breastmilk	‘I'm a breastfeeding peer supporter and feel so passionate about the power of breastmilk that I really wanted to help’.
	Normalizing milk donation	‘I think donating breast milk should be normalized’.
	Dislike of the formula industry	‘The idea of formula companies profiting from unwell and tiny babies makes me rage’.
Practical reasons	No room in the freezer	‘My husband wanted space for food in the freezer!’
Encouraged to by others	Health professional	‘As a health care worker I had seen mothers donate milk and seen donor milk used and I always knew if I had a good supply I would want to do this too’.
	Experience of friends or family	‘My husband was a premature baby and his mum always mentioned how hard it was trying to express milk for him and then not being able to breastfeed’.
	Influence of others	‘My mum is an obstetrician and told me I had to (I wanted to really!)’
Ethical reasons	Moral impediment	‘I felt really lucky to have an excess of milk when others might not. For me I felt like it was morally the right thing for me to do’.
	‘Paying back’ good care	Pay back for the generally good antenatal/post natal care I received for my babies and wonderful care received under Tommy's recurrent miscarriage clinic.
Wanting to help others more broadly	Helping society	During the pandemic I wanted to help and support society in any way I could
	Making the world a better place	‘I watched the news and felt terrible about all the bad in the world. I like the idea of helping and breastmilk feels like the most heart felt of gifts’.
	Unable to donate blood	‘I am unable to donate blood due to health conditions and wanted another way to help others’.

### 3.2 Donation rates

All participants in the study had thought about donating milk and wanted to do so. Overall, 417 (36.3%) went on to donate their milk and 732 (63.7%) did not. For those who did not donate, 391 (53.4%) did not enquire about donation as thought it was not possible. 341 (46.6%) enquired but did not go on to donate. Of those who enquired but did not donate, 218 (63.9%) were told that they could not, 59 (17.3%) decided not to and 64 (18.8%) received no response.

#### 3.2.1 Results from the mothers who were able to donate

*Impact of being able to donate milk*

Participants who were able to donate milk (*n* = 417; 36.3%) were asked how strongly they agreed via a five‐point Likert scale with a series of statements exploring how donation made them feel (Table 4). Feelings of achievement, pride and happiness at helping others were very common. Conversely, challenging feelings such as the experience being hard work or tiring were less common but still present amongst the sample.

**Table 4:** Feelings at being able to donate (strongly agree and agree, *n* = 417).

	*N*	%
I felt that I had achieved something important	412	99.8
Like I was helping other families	406	97.3
Happy that I could help other families	406	97.3
Proud	400	95.9
I felt altruistic	395	41.7
Useful	392	94.0
Like I was paying it forward	329	78.8
Appreciated	324	77.7
Part of a community of mothers	299	71.7
It was hard work to donate milk	154	36.9
I found donating milk tiring	75	18.0
I felt pressure to donate	15	3.6
I incurred costs that I resented	13	3.1

In the open‐ended text, mothers reported that donating their breastmilk was generally a very positive experience. Helping others made them feel good about themselves, with many women expressing that donating had boosted their mood and overall mental health.

‘I felt good about something that I was doing and that would often impact my mood for the rest of the day’. (35, White, North West England)

‘It was one of the most precious gifts that I could offer. It made me very happy to help other babies. I didn't even know them, I didn't see them, but I knew my breastmilk saved lives’. (43, Indian, Wales)

Eight further themes were identified relating to the impact of being able to donate breast milk on their wellbeing.

*Helping to cope with perinatal mental health challenges, trauma and baby loss*. For some mothers, the power of donating breastmilk and its impact on mental health went much deeper, helping them cope with postnatal depression and anxiety, or birth trauma. It gave women a positive task to focus on and feel good about, helping them feel that their body was ‘working’ after it had not in the way they had hoped during birth.

‘I had postnatal depression with my first baby and it helped me feel better about myself as it helped me focus externally on helping others meaning I spent less time inside my head’. (34, White, Wales)

‘I had an awful birth and being able to use my milk positively in this way really helped me… as it felt as if something was going right. Like it was healing almost’. (29, Black, South East England)

Similar feelings arose for mothers who were able to go on to donate after difficult breastfeeding experiences. Donation helped them to create new positive experiences relating to breastfeeding, which was a healing process.

‘Very mentally healing after struggling to breastfeed my first baby’. (30, White, South East England)

‘Definitely helped reduce feelings of depression, sadness and failure in feeding my own baby’. (27, Mixed race, West Midlands)

For mothers who had lost a child, donating breastmilk gave them something positive to focus on and helped them cope during one of the most difficulties times in their lives.

‘Donating milk was hugely beneficial to my mental health following my son's death’. (27, White, North West England England)

‘It also gave, not just myself, but my husband who helped me with cleaning equipment etc some purpose at the worst time in our lives. It gave us a reason to get out of bed and to look after our bodies, when it would have been so easy to rot in bed, angry at the world…it helped to have this gentle, loving place to be while processing the worst time of my life after our son's death’. (37, White, South East England)

*Improving feelings of self‐worth, purpose and identity as a mother*. Donation also helped improve participants sense of self‐worth and self‐identity, during a time when these had been affected by the new challenges of motherhood. It gave mothers some purpose outside of directly caring for a baby, helping them feel ‘useful’ outside of this immediate relationship. Others valued the structure and goal setting it brought.

‘Being able to donate gave me a feeling of worth and that I was able to be useful at a time when I was really struggling to come to terms with the changes associated with motherhood (especially stopping work and not earning a salary ‐ things that had always been really important to my identity)’. (32, White, South East England)

‘It felt good to be working towards something ie collecting enough milk to get it collected by the milk bank’. (33, White, South East England)

Mothers often talked about the feelings of pride and achievement being able to donate milk brought them.

‘I had a real sense of pride and fulfilment’. (32, Asian, Wales)

‘Donating my milk made me feel like I had some sort of control over everything going on it was one thing I felt “good at”’. (31, White, Yorkshire and the Humber)

*Gratitude and ‘paying back’*. For mothers who had experienced breastfeeding challenges and, particularly if they had received DHM for their own baby, being able to donate helped them to ‘pay it back’ into the system. This felt like a way of thanking other women for donating and neonatal intensive care unit (NICU) staff for the support that they received.

‘Donating milk made me feel I was paying back for the milk my baby received after birth’. (30, White, North West England)

‘I was determined to do so and I am so glad I did, as I benefited from donor milk for both my children and I was therefore able to pay the gift forward to other parents in need’. (36, White, South West England)

*Relationship building*. Donation was also described as a means of helping support relationships and attachment with their baby. Mothers felt like they were achieving something as a team with their baby. For others, it was described as a way of connecting them to their own mothers.

‘Really strong relationship with my daughter and what we were achieving’. (32, White, Wales)

‘It connected me with my mum. She donated gallons to the Milton Keynes milk bank in the 70s (my dad would drop it off every Monday morning) and it made me feel connected to her. She would have been proud of me’. (45, White, London)

Connection with their baby was also expressed by some mothers who had experienced baby loss. Being able to donate helped them to honour them.

‘It was a way to honour my son after he died. It was his milk and it meant he was able to have a good impact on the world’. (37, White, South East England)

‘It helped me to feel connected to my baby even though he wasn't here’. (39, White, North West England)

*A greater appreciation of what their body could do*. Some women reported that donating had increased their body image positivity and their appreciation for their own body and what it can do, which in turn improved confidence and wellbeing. For some, this was a feeling of reclaiming their body after a difficult birth or breastfeeding experience, or infant loss.

‘I had a constant feeling that my body had failed me during the birth, but donating milk made a slight difference to my thinking that my body was ‘so broken’ after all’. (34, White, South East England)

‘…donating helped me feel like my body could at least do something that it was actually designed to do and that that was going to help other babies like her’. (38, White, Scotland)

For others, the ability to produce milk not only for their own baby but also for other babies made them feel proud and in awe of their body. It helped them to heal long‐lasting body image issues, helping them to appreciate their female physiology and what it could achieve.

‘Since having children and breastfeeding I have a greater appreciation for what my body can actually do and to know that I not only sustained my own child solely on my breastmilk for 6 months but also to save extra to help other mothers and nourish their babies has had a positive impact on me too’. (42, White, Scotland)

‘It made me feel powerful. The female body is amazing’. (34, White, South East England)

Another aspect of this was realizing how continued milk production and expressing also helped them to protect their own health through reduced risk of maternal cancers.

‘I knew it was protecting my health as well which was a great feeling’. (32, Mixed race, North East England)

*Relief at milk not going to waste*. Some mothers had an excess of milk, because they had expressed more than their baby needed, or their baby would not take a bottle. Being able to donate the milk meant that they felt the time, effort and milk was not going to be wasted.

‘It was a relief not to throw it away and have somewhere to send it’. (35, White, South East England)

‘I had been found crying on the kitchen floor covered in milk, engorged, in pain and on the verge of mastitis several times. I was able to use that milk to hopefully help someone else so had an overwhelming sense of satisfaction’. (40, White, East Midlands)

*Short‐term anxiety or pressure related to the logistics of donation*. Some mothers did identify stressful thoughts or experiences around the experience of donation. This often related to feeling pressure from the different steps of donation, even though overall they wanted to donate. Some had anxieties around making enough milk, expressing it and storing it correctly. Other pressures such as looking after children, housework and other commitments could exacerbate this.

‘I do think it might have increased my anxiety due to wanting to get “enough” milk, store it correctly, avoid medication, fill in sheets, store and transport it, etc. I was already mentally quite fragile and maybe should have donated a bit less and not become so obsessed’. (37, White, North West England)

‘It added an extra chore to a household with 4 children already’.” (30, White, Scotland)

Sometimes milk could not be used due to contamination and this could be very challenging for mothers to hear.

‘I was gutted to hear that the milk bank had thrown away all my milk. If they had tested a small amount and then found it unsuitable then I could have given the rest to someone else in need. Seemed such a shame to waste such hard worked for milk’. (33, White, Scotland)

Additionally, one mother was frustrated that the milk bank had not communicated with her about whether or not her milk had been used.

‘I don't know whether all my milk was used or if any was thrown away’. (32, Mixed race, London)

*Motivation to continue breastfeeding*. Although the survey did not directly ask about breastfeeding experience, several mothers described how the process of donating helped them to feel more motivated to continue breastfeeding.

‘I think it probably motivated me to keep breastfeeding as I was helping others as well as my own baby’. (41, White, Scotland)

‘Yes, on days when I thought I was being a rubbish mum or my baby wasn't getting the best (mainly because he wouldn't latch so I exclusively pumped for him), I reminded myself that I'm not just feeding him’. (32, White, South East England)

#### 3.2.2 Results from mothers who were unable to donate

All participants who did not donate their milk (*n* = 732; 63.7%) responded to a series of statements around how this made them feel. A multivariate analysis of variance found significant differences in emotions between mothers who assumed they could not donate, who were told they could not donate and those who did not receive a response when they enquired about donation (Table 5). Examining the means, typically those who received no response or were told no experienced stronger negative emotions and weaker positive emotions compared to the decided not to donate and did not enquire groups. Posthoc Bonferroni tests identified this pattern for most emotions at *p* < 0.001 and a full set out outputs can be found in Supporting Information S1: Appendix 1.

**Table 5:** Reactions to being unable to donate (showing strongly agree and agree) split by assumption could not donate versus did not donate after milk bank contact.

	Assumed could not (*n* = 391		Told could not donate (*n* = 218		Decided no (*n* = 59)		No response (*n* = 64)		
	*N*	%	*N*	%	*N*	%	*N*	%	Significance
Disappointed	320	81.8	202	92.6	50	84.7	61	95.3	F(3, 723) = 5.86, *p* = 0.01
Frustrated	301	76.9	197	90.3	48	81.3	48	75.0	F(3, 723) = 14.35, *p* ≤ 0.001
Like my milk would be wasted	238	60.8	149	68.3	18	30.5	46	71.8	F(3, 723) = 13.63, *p* ≤ 0.001
Guilty that I couldn't donate	245	62.6	138	63.3	37	62.7	54	84.4	F(3, 723) = 5.96, *p* ≤ 0.001
That breastfeeding wasn't valued	219	56.0	108	49.0	15	25.4	51	79.7	F(3, 723) = 23.10, *p* ≤ 0.001
Upset	218	55.7	145	47.2	36	61.1	50	78.1	F(3, 723) = 8.26, *p* ≤ 0.001
Unfair that I couldn't donate but others could	185	47.3	103	47.3	11	18.6	57	89.1	F(3, 723) = 26.58, *p* ≤ 0.001
Rejected	180	46.0	159	72.9	16	27.1	37	73.5	F(3, 723) = 29.17, *p* ≤ 0.001
Excluded	161	41.1	116	53.2	11	18.6	34	53.1	F(3, 723) = 12.87, *p* ≤ 0.001
Like my milk wasn't good enough	150	38.3	101	46.3	9	15.3	39	59.1	F(3, 723) = 15.884, *p* ≤ 0.001
Happy that I'd tried to donate	NA	NA	140	64.2	23	39.0	23	35.9	F(2, 333) = 11.80, *p* ≤ 0.001
Relieved I would not be committing	75	19.1	20	9.2	22	34.3	5	7.8	F(3, 723) = 13.38, *p* ≤ 0.001
Ambivalent, it didn't matter to me	42	10.7	14	6.4	4	6.8	0	0.0	F(3, 723) = 5.29, *p* = 0.01

Abbreviation: NA, not applicable.

Thematic analysis identified six themes related to the impact of being unable to donate upon wellbeing. Feelings of disappointment, frustration, sadness, rejection and anger were common, with some still feeling the intensity many years later.

‘I still feel that sadness now and she's seven’. (42, White, West Midlands, did not enquire)

‘I'm still angry over a decade later’. (50, White, London, told no)

*Disappointment and sadness*. Many mothers expressed their disappointment and sadness at not being able to donate as they felt they had missed out on the opportunity to help other mothers and babies, which was important to them. This could be particularly difficult if they were already having a challenging time postnatally or had experienced birth difficulties.

‘It hurt because I wanted to help others and I wasn't allowed. Even though this wasn't my fault it stung. I didn't need any further hurt at that time and came to regret asking and wanting to do it if that was the outcome’. (33, White, Yorkshire and the Humber, told no)

‘I really hoped it would be possible and it wasn't and it broke my heart a little bit all over again’. (37, White, East Midlands, told no)

*Rejection*. Closely linked to feelings of sadness were those of rejection. Mothers felt that their milk wasn't good enough and as if they were being rejected or discriminated against when others had the opportunity.

‘Annoyed that there wasn't a level of scrutiny with the impact of the medication on the breastmilk. Just felt it was written off before anyone really weighed the risks and benefits. I felt really rejected and almost discriminated against’. (31, White, North West England, told no)

‘It made me feel like I was sick or tainted, when I don't usually feel that way’. (33, white, South East England, told no)

For some, this made them question the quality of their milk, or how human milk was valued. It could be particularly difficult to be told that it was fine to breastfeed their own baby but not to donate milk.

‘I was confused why my milk was good enough for my baby but not for others’. (33, White, Northern Ireland, told no)

‘It made me question if I should be breastfeeding my baby if my milk wasn't suitable’. (38, White, South West England, told no)

*Frustration and anger*. Frustration and anger were common emotions at not being able to donate. Many expressed frustration with the milk bank rules feeling that they were too strict about the age of babies or the processes to set up donating being too complex. Others expressed anger that the infrastructure was not in place to be able to transport their milk.

‘There was a shortage of syringes for blood tests [due to Covid‐19] and by the time my GP allowed blood tests my baby was 7 months so I missed the milk bank cut off of 6 months. I was frustrated and confused’. (33, Chinese, South East England, told no)

‘I was angry that there was no milk bank close enough’. (31, Black, Wales, told no)

This included anger for themselves, when they already had gone through the process of expressing and storing milk for the milk bank, and they felt they had wasted their time and effort.

‘I had 60 oz pumped and frozen in total. I cried when I was putting it in the bin’. (24, White, South East England, told no)

‘It is heartbreaking. I have two drawers full of breast milk in my freezer and all of them will now end up in the bin. We are going abroad for four months so we won't be able to use the milk in the freezer within the six month period’. (35, Asian, South East England, told no)

Some felt anger that that more babies were not able to benefit from available breastmilk, knowing that there were restrictions on access. A common part of this frustration was not understanding why potential risks of not receiving human milk were not considered and why formula was seen to be safer.

‘I felt angry that the bank was only providing milk to certain babies and wasn't big enough to provide to more. We know the impact on health of very small amounts of formula even as a temporary measure, those needs felt dismissed and parents uncared for. I was more angry for parents who would be grateful but their babies were “too old” or “too healthy”’. (33, White, South West England, told no)

‘It felt like such a waste. I knew how important breastmilk was for premature babies and I had loads of it but couldn't give it to a milk bank. It seemed illogical. I knew they needed milk so why wasn't there a system in place to reach women like me? It's not like I live miles from civilization! It really upset me and in a way felt like I was being deprived of an opportunity to help?’ (45, White, Wales, did not enquire)

These feelings were also common amongst those who received no response.

‘Frustrating that no one replied given I could help them’ (31, Mixed race, West Midlands, no response)

‘It was very important to me and it upset me a great deal that no one got back to me’. (34, White Irish, Wales, no response)

*Guilt at not being able to ‘pay it back’*. Alongside feelings of frustration, mothers whose own baby had received donor milk but who were unable to donate often felt guilt at not being able to support others.

‘My baby had donor milk but I couldn't pay it forward and that upset me’. (43, Mixed race, Wales, told no)

‘I wanted to donate so badly as I received milk for my daughter and wanted someone else to have that opportunity, like I was paying it forward. I was gutted that I couldn't as never expected them to say no. I felt guilty in a way even though I know it wasn't my fault really’. (27, White, Wales, told no)

Guilt, at wasting time and resources, was also experienced by some mothers who were able to start the process of donating their milk but struggled to express enough milk.

‘I felt guilty that I had cost the milk bank money by having the blood tests and all the bottles sent to me but I couldn't donate enough’. (41, White, South East England, decided not)

‘I felt guilty that I had been through the motions and started to collect and had all of the bottles etc sent through but didn't manage to express enough milk with my hand pump/find the time to do it each day’. (35, White, South East England, decided not)

*A missed healing opportunity*. As described in the section above about the impacts of being able to donate milk, donation could play an important role in helping to heal trauma, particularly for those who had lost a baby. Sadly, several women who had experienced baby loss were then unable to donate their milk, which exacerbated the pain further, denying them of that outlet.

‘Incredibly sad that I had milk that my stillborn baby couldn't have but another baby could have benefitted from’. (44, White, South West England, told no)

‘I really wanted my milk supply to be of use as my baby had died. I found expressing so easy, had the right storage too’. (29, White, South East England, told no)

Others felt that they had been denied an opportunity to heal, or that difficult emotions had been further exacerbated, after breastfeeding difficulties or birth trauma.

‘My own baby had been very sick after birth and I really wanted to help. It made me feel really unsettled that I couldn't because no one was answering’. (34, White, London, no response)

‘At the time I took it really personally. I'd had a traumatic birth and managed to breastfeed after desperately asking for help but getting very little NHS support (due to COVID restrictions) so I was proud of having enough to be able to help others but couldn't’. (30, White, East Midlands, told no)

*Relief at not having to go through the process*. A few women reported mixed feelings about not being able to donate, expressing sadness or disappointment but also relief as they didn't have to find the time to pump extra milk.

‘Slightly frustrated as I know how much I'd have liked to use donor milk if I'd been unable to BF, glad I tried, but frustrated I couldn't but simultaneously relieved I didn't have an extra thing to do’. (30, White, East Midlands, told no)

‘After a while I realized it might have worked out for the best, as breastfeeding and pumping took up a lot of time and to add in extra pumping might have been too much in the end’ (35, Mixed race, London, told no)

## 4 DISCUSSION

This study highlights the perceived self‐reported impacts upon wellbeing and mental health of mothers in the United Kingdom who were able to donate their milk to a milk bank or not. Mothers who were able to donate described how the experience helped them to feel proud, useful and that they were achieving something. Conversely, those who were unable to donate often felt rejected, frustrated and excluded. These emotions could be heightened amongst mothers who had experienced baby loss, breastfeeding difficulties, birth trauma or had received donor milk for their own baby in neonatal care. For those who could, being able to donate helped to heal these experiences, whereas for others being prevented from doing so could exacerbate feelings of loss. Although some mothers in the study would have been unable to donate their milk, some were prevented from doing so due to logistical reasons and the findings therefore have important implications for considering service expansion.

The findings echo many of the themes that arise in broader research exploring maternal wellbeing and infant feeding experiences. When women were able to donate their milk, this enhanced feelings of pride, achievement and usefulness, supporting maternal identity and mother–infant bonding. These themes reflect previous research into the impact of milk donation (Candelaria et al., 2018; Doshmangir et al., 2019; Miranda et al., 2016; Olsson et al., 2021; Wambach et al., 2019) but also mirror the emotions around broader breastfeeding experiences. When women feel supported, respected and enabled to meet their infant feeding goals, this can boost maternal mental health in similar ways (Brown, 2018), particularly when babies and children are premature or unwell (Avilla et al., 2020; Flacking et al., 2016; Hookway et al., 2023; Shepherd et al., 2017).

Within this, it was notable how being able to donate milk was often described as giving mothers a sense of purpose at a difficult time. The perinatal period is a time of great change and can feel overwhelming as women start to adapt to new motherhood and their new identity (Winson, 2017). This can be particularly difficult for women who have established careers and working identities outside of the home (Feng & Savani, 2020). New motherhood in contrast can feel like a challenging and lonely experience (Lee et al., 2019) coming as a culture shock (Huppatz, 2018). Some mothers attributed being able to donate milk as helping them to navigate these feelings, ‘pulling them out’ of depressive or anxious thoughts because of the sense of purpose and focus outside of themselves.

There was an interesting contrast between these feelings of achievement, value and appreciation at donating milk and the opposite feelings of dismissal and insignificance that breastfeeding women can feel (Taylor et al., 2019; Tomori et al., 2016). There are parallels to perceptions of mothers and work; women describe how the effort of caring for their own baby feels as if it is dismissed yet placing their baby in childcare while they work is valued (Pedersen, 2016). Calculations of how much breastmilk is worth globally to the economy through unpaid care work impacting upon health, development and productivity have highlighted its worth (Smith et al., 2023). Women may view their donation of milk to others as a valued ‘gift’ but it seems that the inherent value of human milk (and the physical and emotional labour involved in producing it) is yet to be fully appreciated in society, both in relation to donated milk and to breastfeeding one's own baby (Cassidy, 2012).

The experience of being able to donate milk also helped to support mothers who had experienced a difficult birth or early breastfeeding experience to feel that their body was now ‘working’. In contrast to feelings around birth, donation could bring about a sense of purpose and control; mothers set out to produce, express and store milk, and achieved doing so. This group of mothers may also be more aware of how challenging birth and early feeding experiences can be and are motivated to help others during these difficult times. Particularly for those who had struggled to establish a supply themselves, being able to not only establish feeding but go on to support others was important. This reflects previous research describing how breastfeeding can be part of the healing process from birth trauma, as it can help women regain feelings of control and that their body is working in the way that they hoped (Beck, 2022; Brown, 2018; Kendall‐Tackett, 2014).

This closely fitted with the experiences of those who had received milk for their own premature baby in NICU. Donating was about ‘paying it back’ and supporting the system that had helped them, consistent with previous research (Cassidy & Dykes, 2019; Wambach et al., 2019). Mothers in this group were aware of how distressing not being able to make enough milk could be and wanted to help others avoid this (although it should be noted that recipient mothers should also receive support to build their own supply where possible to reduce risk of supplementation potentially interfering with milk production longer term). This echoes previous research with donor milk recipients who felt increased motivation to build their own supply through challenging experiences, with the hope of supporting others (Brown & Shenker, 2022; Brown et al., 2024). Donation was also particularly helpful for those who wanted to donate after baby loss, helping them feel that their baby ‘lived on’ and was helping to support other families. The experience of expressing and donating milk also helped them to feel connected to their baby, echoing previous research (Cassidy & Dykes, 2019; Cole et al., 2018; Oreg, 2020; Welborn, 2012).

Conversely, when donation was not possible, this could lead to feelings of disappointment, frustration, guilt and anger, mirroring complex emotions mothers can feel when they experience breastfeeding difficulties or have to stop breastfeeding before they are ready (Brown, 2018, 2019; Jackson et al., 2021; Thomson et al., 2015). Feelings of rejection and exclusion from donation may also mirror similar feelings of women unable to breastfeed who report upset at not being able to be part of a ‘group’, that is, not being able to be part of an exclusive breastfeeding community (Brown, 2019).

Not being able to donate was particularly difficult for those experiencing birth trauma or postnatal depression and anxiety. It exacerbated feelings of ‘failure’, frustration and felt like a lost opportunity to heal (Chatzopoulou et al., 2023) and felt like a further rejection akin to feelings women can experience around a lack of support for breastfeeding difficulties (Lawlor et al., 2023; Regan & Brown, 2019). Again, in contrast to positive experiences of donation, not being able to ‘pay it back’ after receiving milk for a baby in NICU or benefit from being supported to donate after baby loss could exacerbate distress. Although these mothers made up just a small part of our sample (*n* = 19, 1.9%), this presents a key point for potential action. Previous research has identified how donation after loss is rarely discussed, with mothers often offered support with lactation suppression rather than donation (Britz & Henry, 2013; Carroll et al., 2020; Sweeney et al., 2020). Lactation suppression can be a challenging experience, exacerbated for bereaved mothers who have to navigate physical discomfort such as breast engorgement and leakage during the days after infant loss (McGuinness et al., 2013). This work presents an important argument for donation as an alternative, providing relief from physical discomfort as well as potentially becoming an avenue for healing.

Clearly, milk banking regulations are in place to support the health of the most vulnerable infants (Hartmann, 2017). Milk donation is not always possible for women, with milk banks typically having criteria around amount of milk available, distance from the milk bank (due to the logistical need to transport milk and keep it frozen during transit) and health considerations or lifestyle factors based on NICE guidelines (NICE, 2010). Some of these issues will not be modifiable, that is, certain medications are contraindicated as they pass into breastmilk in significant amounts. However, our findings show that many more women could be supported to donate their milk with increased investment into infrastructure (more local storage hubs and transport options) and staffing to meet increased demand, including support to respond to all queries. Increased willingness to expand donor milk use in hospitals is also growing (Shenker et al., 2023). Yet, currently infrastructure and lack of investment are some of the key barriers to expansion and sustainable provision (Kaech et al., 2022; Mathias et al., 2023), particularly at times of crisis such as the Covid‐19 pandemic (Shenker et al., 2020, 2021).

There are also variations in factors such as age limits of infant at the start of a donation period or a donor's proximity to a milk bank or hub which need consideration, especially with more investment into research. Research into the content of milk during later infancy suggests subtle changes that may need to be taken into consideration. For example, one longitudinal study examining content of milk between 11 and 17 months postpartum found a significant increase in some factors such as total protein, lactoferrin, lysozyme, Immunoglobulin A, oligosaccharides and sodium but a decline in zinc and calcium concentrations. There were no changes for lactose, fat, iron and potassium. Comparing it to donated milk from mothers with a baby under 1 year, milk in the second year contained significantly higher concentrations of total protein, lactoferrin, lysozyme and Immunoglobulin A, and significantly lower concentrations of zinc, calcium, iron and oligosaccharides. The authors suggest that accepting milk after 1 year postpartum would expand supply, but consideration is needed as to whether it would need mineral fortification (Perrin et al., 2017).

Alongside this, further education and promotion of milk banking criteria would be beneficial in supporting women to enquire about milk donation at an earlier stage. This could avoid losing mothers from the system because enquiries were made when babies were older, or milk had not been stored correctly. Increased awareness is also needed around why donor milk restrictions for medication and other lifestyle factors are in place, especially when women are told they cannot donate but can continue to breastfeed their own baby. This is typically due to the need for stricter standards due to most donor milk being received by vulnerable, sick and premature infants. However, it is possible that if donor milk criteria are expanded in future to offer it outside of prematurity (e.g., for additional feeds for term hypoglycaemic infants instead of formula milk), then it may be possible to expand who can donate (Griffin et al., 2022; McCune & Perrin, 2021). Further research is needed into which medications are contraindicated or may be possible in different scenarios.

However, a core point to consider when reflecting on how to increase donor milk supplies and to support more families with their feeding preferences, is to focus our attention on how greater support is given to women with breastfeeding and providing their baby with human milk in the first place. The last infant feeding survey in the United Kingdom in 2010 found that just 1% of women exclusively breastfed for 6 months, with more than half of babies receiving formula by the end of their first week of life. Exclusive breastfeeding rates at 3 months of age were just 15% (McAndrew et al., 2012). Although breastfeeding rates may be increasing, and some women will make the decision not to breastfeed or be unable to do so, for many women in the United Kingdom their ability to breastfeed is hampered by a wider lack of support across health services, family and in public spaces (Brown, 2017). This can be particularly true for those with a baby in neonatal care and can be highly dependent on the depth and quality of support that mothers receive (Hilditch et al., 2024). Ensuring that women and families receive high‐quality breastfeeding support in the first place would increase breastfeeding rates and exclusivity, thus protecting maternal and infant health and wellbeing (Brown, 2018), but also reduce the need for donor milk, enhance the number of women who are able to donate and enable more babies who cannot receive their mother's own milk to receive human milk.

Alongside this, it is also important to consider how mothers who are unable to donate may be better supported. Being able to donate could elicit feelings of pride and achievement but when mothers were not able to do so, this denied them this opportunity and risked instead eliciting negative emotions. Recognizing the current time and resource limits on milk banks, it may be useful to reflect on whether additional support could be developed for those who are unable to donate, particularly for women who are driven to donate through their own difficult experiences such as previously having a baby in neonatal care or infant loss. As above, more detailed information around decisions may be useful but there is also a gap to develop support for mothers to discuss their experience of wanting to donate and not being able to. Similarly, how could we better support mothers to gain a feeling of pride and altruism through their willingness to donate even if unable to do so?

Potentially this service could be part of support provided by breastfeeding organizations who already provide counselling support around infant feeding difficulties and breastfeeding grief. Further research might wish to explore, with mothers, what would have helped them process this decision and how they feel about their breastfeeding experience.

It may also be useful for more information and options to be available for women to help milk banking services or breastfeeding support in other ways. Part of the reasons why women wanted to donate, or the positive feelings they experienced around being able to do so were that they felt useful that they were making a difference or were giving back to the service. Might volunteering, wider advocacy or supporting donation help to elevate similar experiences?

Some mothers who donated did experience challenges or felt that the process was at times difficult, reflecting findings from other studies (Hyde et al., 2023; Olsson et al., 2021; Wambach et al., 2019). This does not mean that donation had a negative impact on their wellbeing, but rather they experienced mixed emotions, yet still made the decision to continue to donate. This has interesting parallels to how women sometimes feel about breastfeeding. Challenges can be common, or women might feel exhausted by frequent feeding, yet do not wish to stop (Brown, 2018; Fox et al., 2015). However, tendencies to describe breastfeeding as ‘easy’ can lead to mothers feeling unprepared and portray the idea that something needs to be easy to pursue (Brown, 2016). The same may apply to discussions around milk donation, with discussions taking a pragmatic approach to consider some of the potential challenges so they are not felt to be uncommon or unexpected.

A small number of women in the study felt that donating milk increased their anxiety, feeling pressure to make ‘enough’ milk or worrying about contamination. Awareness of the potential for the process to make existing postnatal anxiety worse is needed alongside strategies to balance risk avoidance with reassurance. This is why lactation support alongside donation is so important and emphasizes the need for investment in training for milk bank staff (Griffin et al., 2022). Additional training is needed to support communication of women who either cannot donate or who have repeated microbiological fails of their milk, both to support their wellbeing and to protect on‐going breastfeeding of their own infant.

The relationship between milk banks and milk‐sharing communities is highly relevant to this work. A recent survey of milk donors to milk banks in the United States and United Kingdom highlighted a significant proportion of women (51% in the United States and 39% in the United Kingdom) went on to share their milk in the community, mostly to families they didn't know, or to friends and family, as well as donating their milk to a milk bank (Dos Santos et al., 2024). Interestingly, sharing milk with others was associated with a longer donation period for milk donors and, in the United States, greater donation volumes. These findings counter concerns from milk banks that sharing milk might decrease the donors’ commitment to milk donation (Emba & Hmbana, 2015) and may reflect additional feelings of positivity associated with providing more broad support for others or the reinforcement of previously held attitudes around the significance of human milk (Palmquist et al., 2019; Perrin et al., 2016).

Currently, there are no mandatory blood tests or screening processes for women who engage in milk sharing and information for recipient parents about accepting shared unscreened milk may not be adequate for them to be fully informed and aware of risks that could be mitigated by donor screening or home‐based pasteurization. It would be important to understand in future studies the drivers for those women who both donate and share their milk compared to those who only donate, share, or sell their milk. Furthermore, milk banks could provide a useful interim service to provide not only evidence‐based information for milk‐sharing communities but also a mechanism through which donors could undergo serological screening. However, a full assessment would need to take into account the costs incurred as a result to milk banks, time commitment of milk bank teams that are already overstretched and the question of where future liability would lie in the case of any harm to the recipient infant as a result of milk‐sharing activity.

Only 2% of milk donors in the survey of US‐ and UK‐based milk donors also reported selling their milk to a person or company, which was in accordance with previously reported data from 2018 (Dos Santos et al., 2024; O'Sullivan et al., 2019). Despite well‐recognized harms of the commercialization of other biofluids (Infected Blood Inquiry, 2024; Rogowska‐Szadkowska, 2011), the sale of human milk has generally been little discussed, despite the risk to the provider of legal action if harm came to the recipient or the milk was contaminated with another mammalian species’ milk (David, 2011). These harms have been enshrined in the European Union Directive on Blood, Tissues and Cells (Directive 2004/23/EC), which states the human body should not be commercialized or the source of gain. The recent update to this directive has for the first time included DHM as a Substance of Human Origin (SoHO) (European Commission, 2024) (REF). Although the regulation of DHM currently differs between countries (Shenker et al., 2024), there are now clear rationales for countries globally to legislate for DHM as a SoHO, with the aim of limiting the potential harms of commercialization, including those that may affect the donor (Herson & Weaver, 2024; Klotz et al., 2022).

Finally, our findings also add to the growing body of evidence which has illustrated the negative impact that the Covid‐19 pandemic and restrictions had for some breastfeeding women, particularly for those with a baby in neonatal care (Brown & Shenker, 2022; Pacheco et al., 2021; Spatz et al., 2021). Some women highlighted their distress at how their breastfeeding experience had been disrupted by reductions in being able to seek face‐to‐face support, which were then compounded by not being able to donate. Others were impacted by shortages of vials or limits on blood tests delaying the enrolment process meaning that their baby was then too old for them to donate. This reflects experiences of many milk banks globally, where restrictions, shortages and fears around the virus reduced the number of women who were ultimately able to donate. Recommendations have been made as to how this could be avoided in future similar scenarios such as increasing support to mothers, adapting strategies to ensure testing and collection can take place, and increasing awareness and engagement in the need to maintain and increase stocks at this time (Shenker et al., 2020, 2021).

Our study does have limitations. Our participants were older than average with a higher level of education and self‐selected into the research although this reflects similar demographics in other hospital‐based research in infant feeding (e.g., Flaherman et al., 2019; Kair & Flaherman, 2017; Soltani & Scott, 2012). This may have been exacerbated by internet‐based recruitment, although the use of smartphones and social media in this age cohort is high and unlikely to restrict access any more than known limitations with face‐to face and hospital‐based recruitment. Internet recruitment also allowed a wider geographic spread of participants to be accessed and to reach those who were unable to donate milk (i.e., would not have been part of any milk bank register). Our convenience sample participant split of one‐third who were able to donate and two‐thirds who did not, should not necessarily be considered representative of UK donation rates. It is likely that those who had positive or negative donation experiences were more likely to take part. It would be interesting to audit how many women who contact each milk bank in the United Kingdom are able to donate although this would exclude those who did not realize that they could donate or who did not receive a response. It would also be interesting to compare and contrast this issue internationally. Although donor attributes and beliefs have been reviewed across countries (Dos Santos & Perrin, 2022), it would be useful to consider how milk banking infrastructure, investment and attitudes towards donation affect ability of women to be able to donate.

However, disparities are highlighted in who is able to enquire about milk donation. In the United Kingdom, mothers who breastfeed for a longer duration are typically older and have a higher level of education (McAndrew et al., 2012), with milk donors from high‐income countries also following this pattern (Dos Santos & Perrin, 2022; Kundisova et al., 2019). Although participants from multiple ethnic groups took part, only around 6% of our sample were from Black, Asian and mixed‐race backgrounds compared to a national population of 14%. Some mothers from Muslim families may not wish to donate (or receive) milk due to milk kinship: an Islamic belief that human milk creates a kinship between the breastfeeding mother's family, including her children, and the infants who receive her milk. This relationship prevents marriage in Islamic law and the anonymity between donor and recipient in milk banking creates a potential issue (Khalil et al., 2016), which can be overcome through accurate systems of traceability.

Although historically all communities have practiced wet nursing to build bonds, support families and to protect infants, there have also been periods when wet nursing has been used harmfully, such as enslaved women being forced to wet nurse. This has particular implications within Black communities in relation to milk donation, exacerbated by recent events such as for‐profit milk banks in the United States specifically targeting Black mothers to sell their milk (Harrison, 2019). Further culturally informed research will be needed to explore and navigate these and other barriers to milk donation before true equity can be achieved.

In conclusion, our research extends the sample size of previous research, specifically focusing on wellbeing and the emotional experience of donation in a UK sample. It compares the experiences of both those who were able to donate or not donate across a variety of contexts. The findings have important implications for how future donors are supported, milk bank training and the scaling up of milk banking services.

## AUTHOR CONTRIBUTIONS

Amy Brown was responsible for study conception, study design, data collection, data analysis and draft report writing. Catrin Griffiths was responsible for data analysis and draft report writing. Sara Jones, Natalie Shenker and Gillian Weaver were responsible for study conception, study design and draft report writing. All authors read and approved the final manuscript.

## CONFLICT OF INTEREST STATEMENT

Amy Brown coordinates the Swansea Human Milk Bank hub, a collaborative project between the Human Milk Foundation, Swansea University, and Swansea Bay University Health Board, set up of which was funded through Research Wales Innovation Funding from HEFCW. This grant also funded the development of this research. Natalie Shenker is a cofounder and consultant for the Human Milk Foundation, a charity that operates the Hearts Milk Bank. She is a UKRI Future Leaders Fellow at Imperial College London (grant p76489) and UKRI funding supported the writing and publication of this manuscript. Gillian Weaver is a cofounder and consultant for the Human Milk Foundation, a charity that operates the Hearts Milk Bank. Catrin Griffiths has no conflict of interest to declare. Sara Jones has previously worked as an assistant co‐ordinator at the Swansea Human Milk Bank hub.

## Supporting information

Supporting Information

## Data Availability

The data that support the findings of this study are available on request from the corresponding author. The data are not publicly available due to privacy or ethical restrictions.
