# Supplementary material for: Disparities in being able to donate human milk impacts upon maternal wellbeing: Lessons for scaling up milk bank service provision
Source: Matern Child Nutr. 2024 Jul 10;20(4):e13699. doi: 10.1111/mcn.13699 (PMC11574679; doi:10.1111/mcn.13699)
Supplement: Supplementary file 1 — Supporting Information [file MCN-20-e13699-s001.docx]

**Appendix one: Post hoc Bonferroni tests for emotions at not being able to donate**

|  | | | | | | | |
| --- | --- | --- | --- | --- | --- | --- | --- |
| Dependent Variable | Donor Group | Donor Group | Mean Difference | Std. Error | Sig. | 95% Confidence Interval | |
|  |  |  |  |  |  | Lower Bound | Upper Bound |
| How did not being able to donate make you feel? - Rejected | Told no | Decided no | 1.15^*^ | .154 | <.001 | .74 | 1.56 |
|  |  | No response | -.04 | .148 | 1.000 | -.43 | .35 |
|  |  | Didn’t ask | .60^*^ | .088 | <.001 | .36 | .83 |
|  | Decided no | Told no | -1.15^*^ | .154 | <.001 | -1.56 | -.74 |
|  |  | No response | -1.19^*^ | .189 | <.001 | -1.69 | -.69 |
|  |  | Didn’t ask | -.55^*^ | .147 | .001 | -.94 | -.16 |
|  | No response | Told no | .04 | .148 | 1.000 | -.35 | .43 |
|  |  | Decided no | 1.19^*^ | .189 | <.001 | .69 | 1.69 |
|  |  | Didn’t ask | .64^*^ | .140 | <.001 | .27 | 1.01 |
|  | Didn’t ask | Told no | -.60^*^ | .088 | <.001 | -.83 | -.36 |
|  |  | Decided no | .55^*^ | .147 | .001 | .16 | .94 |
|  |  | No response | -.64^*^ | .140 | <.001 | -1.01 | -.27 |
| How did not being able to donate make you feel? - Frustrated | Told no | Decided no | .37^*^ | .128 | .024 | .03 | .71 |
|  |  | No response | -.26 | .123 | .224 | -.58 | .07 |
|  |  | Didn’t ask | .35^*^ | .073 | <.001 | .15 | .54 |
|  | Decided no | Told no | -.37^*^ | .128 | .024 | -.71 | -.03 |
|  |  | No response | -.63^*^ | .157 | <.001 | -1.04 | -.21 |
|  |  | Didn’t ask | -.02 | .122 | 1.000 | -.34 | .30 |
|  | No response | Told no | .26 | .123 | .224 | -.07 | .58 |
|  |  | Decided no | .63^*^ | .157 | <.001 | .21 | 1.04 |
|  |  | Didn’t ask | .60^*^ | .117 | <.001 | .30 | .91 |
|  | Didn’t ask | Told no | -.35^*^ | .073 | <.001 | -.54 | -.15 |
|  |  | Decided no | .02 | .122 | 1.000 | -.30 | .34 |
|  |  | No response | -.60^*^ | .117 | <.001 | -.91 | -.30 |
| How did not being able to donate make you feel? - Upset | Told no | Decided no | .34 | .148 | .137 | -.05 | .73 |
|  |  | No response | -.31 | .143 | .168 | -.69 | .06 |
|  |  | Didn’t ask | .27^*^ | .085 | .008 | .05 | .50 |
|  | Decided no | Told no | -.34 | .148 | .137 | -.73 | .05 |
|  |  | No response | -.65^*^ | .182 | .002 | -1.13 | -.17 |
|  |  | Didn’t ask | -.06 | .141 | 1.000 | -.44 | .31 |
|  | No response | Told no | .31 | .143 | .168 | -.06 | .69 |
|  |  | Decided no | .65^*^ | .182 | .002 | .17 | 1.13 |
|  |  | Didn’t ask | .59^*^ | .135 | <.001 | .23 | .94 |
|  | Didn’t ask | Told no | -.27^*^ | .085 | .008 | -.50 | -.05 |
|  |  | Decided no | .06 | .141 | 1.000 | -.31 | .44 |
|  |  | No response | -.59^*^ | .135 | <.001 | -.94 | -.23 |
| How did not being able to donate make you feel? - Like my milk wasn’t good enough | Told no | Decided no | 1.08^*^ | .200 | <.001 | .55 | 1.61 |
|  |  | No response | -.58^*^ | .193 | .017 | -1.09 | -.07 |
|  |  | Didn’t ask | .09 | .115 | 1.000 | -.21 | .40 |
|  | Decided no | Told no | -1.08^*^ | .200 | <.001 | -1.61 | -.55 |
|  |  | No response | -1.66^*^ | .246 | <.001 | -2.31 | -1.01 |
|  |  | Didn’t ask | -.99^*^ | .191 | <.001 | -1.49 | -.49 |
|  | No response | Told no | .58^*^ | .193 | .017 | .07 | 1.09 |
|  |  | Decided no | 1.66^*^ | .246 | <.001 | 1.01 | 2.31 |
|  |  | Didn’t ask | .67^*^ | .183 | .002 | .19 | 1.15 |
|  | Didn’t ask | Told no | -.09 | .115 | 1.000 | -.40 | .21 |
|  |  | Decided no | .99^*^ | .191 | <.001 | .49 | 1.49 |
|  |  | No response | -.67^*^ | .183 | .002 | -1.15 | -.19 |
| How did not being able to donate make you feel? - Like my milk would be wasted | Told no | Decided no | 1.09^*^ | .191 | <.001 | .59 | 1.60 |
|  |  | No response | -.28 | .184 | .799 | -.76 | .21 |
|  |  | Didn’t ask | .15 | .110 | 1.000 | -.14 | .44 |
|  | Decided no | Told no | -1.09^*^ | .191 | <.001 | -1.60 | -.59 |
|  |  | No response | -1.37^*^ | .234 | <.001 | -1.99 | -.75 |
|  |  | Didn’t ask | -.95^*^ | .182 | <.001 | -1.43 | -.47 |
|  | No response | Told no | .28 | .184 | .799 | -.21 | .76 |
|  |  | Decided no | 1.37^*^ | .234 | <.001 | .75 | 1.99 |
|  |  | Didn’t ask | .42 | .174 | .092 | -.04 | .88 |
|  | Didn’t ask | Told no | -.15 | .110 | 1.000 | -.44 | .14 |
|  |  | Decided no | .95^*^ | .182 | <.001 | .47 | 1.43 |
|  |  | No response | -.42 | .174 | .092 | -.88 | .04 |
| How did not being able to donate make you feel? - Relieved that I would not be committing to something extra to do | Told no | Decided no | -.77^*^ | .158 | <.001 | -1.19 | -.35 |
|  |  | No response | .10 | .152 | 1.000 | -.30 | .51 |
|  |  | Didn’t ask | -.40^*^ | .091 | <.001 | -.64 | -.16 |
|  | Decided no | Told no | .77^*^ | .158 | <.001 | .35 | 1.19 |
|  |  | No response | .87^*^ | .194 | <.001 | .36 | 1.39 |
|  |  | Didn’t ask | .37 | .151 | .090 | -.03 | .77 |
|  | No response | Told no | -.10 | .152 | 1.000 | -.51 | .30 |
|  |  | Decided no | -.87^*^ | .194 | <.001 | -1.39 | -.36 |
|  |  | Didn’t ask | -.51^*^ | .144 | .003 | -.89 | -.12 |
|  | Didn’t ask | Told no | .40^*^ | .091 | <.001 | .16 | .64 |
|  |  | Decided no | -.37 | .151 | .090 | -.77 | .03 |
|  |  | No response | .51^*^ | .144 | .003 | .12 | .89 |
| How did not being able to donate make you feel? - Happy that I’d tried to donate | Told no | Decided no | .38^*^ | .134 | .026 | .03 | .74 |
|  |  | No response | .62^*^ | .129 | <.001 | .28 | .96 |
|  |  | Didn’t ask | .71^*^ | .077 | <.001 | .50 | .91 |
|  | Decided no | Told no | -.38^*^ | .134 | .026 | -.74 | -.03 |
|  |  | No response | .23 | .165 | .941 | -.20 | .67 |
|  |  | Didn’t ask | .32 | .128 | .071 | -.02 | .66 |
|  | No response | Told no | -.62^*^ | .129 | <.001 | -.96 | -.28 |
|  |  | Decided no | -.23 | .165 | .941 | -.67 | .20 |
|  |  | Didn’t ask | .09 | .123 | 1.000 | -.23 | .41 |
|  | Didn’t ask | Told no | -.71^*^ | .077 | <.001 | -.91 | -.50 |
|  |  | Decided no | -.32 | .128 | .071 | -.66 | .02 |
|  |  | No response | -.09 | .123 | 1.000 | -.41 | .23 |
| How did not being able to donate make you feel? - Curious about why there wasn’t a local milk bank | Told no | Decided no | .80^*^ | .152 | <.001 | .40 | 1.21 |
|  |  | No response | .07 | .146 | 1.000 | -.32 | .46 |
|  |  | Didn’t ask | -.18 | .087 | .234 | -.41 | .05 |
|  | Decided no | Told no | -.80^*^ | .152 | <.001 | -1.21 | -.40 |
|  |  | No response | -.73^*^ | .186 | <.001 | -1.23 | -.24 |
|  |  | Didn’t ask | -.98^*^ | .145 | <.001 | -1.37 | -.60 |
|  | No response | Told no | -.07 | .146 | 1.000 | -.46 | .32 |
|  |  | Decided no | .73^*^ | .186 | <.001 | .24 | 1.23 |
|  |  | Didn’t ask | -.25 | .139 | .425 | -.62 | .12 |
|  | Didn’t ask | Told no | .18 | .087 | .234 | -.05 | .41 |
|  |  | Decided no | .98^*^ | .145 | <.001 | .60 | 1.37 |
|  |  | No response | .25 | .139 | .425 | -.12 | .62 |
| How did not being able to donate make you feel? - Excluded | Told no | Decided no | 1.06^*^ | .174 | <.001 | .60 | 1.53 |
|  |  | No response | .06 | .168 | 1.000 | -.38 | .51 |
|  |  | Didn’t ask | .25 | .100 | .078 | -.02 | .51 |
|  | Decided no | Told no | -1.06^*^ | .174 | <.001 | -1.53 | -.60 |
|  |  | No response | -1.00^*^ | .214 | <.001 | -1.57 | -.44 |
|  |  | Didn’t ask | -.82^*^ | .166 | <.001 | -1.25 | -.38 |
|  | No response | Told no | -.06 | .168 | 1.000 | -.51 | .38 |
|  |  | Decided no | 1.00^*^ | .214 | <.001 | .44 | 1.57 |
|  |  | Didn’t ask | .19 | .159 | 1.000 | -.23 | .61 |
|  | Didn’t ask | Told no | -.25 | .100 | .078 | -.51 | .02 |
|  |  | Decided no | .82^*^ | .166 | <.001 | .38 | 1.25 |
|  |  | No response | -.19 | .159 | 1.000 | -.61 | .23 |
| How did not being able to donate make you feel? - Ambivalent, it didn’t matter to me | Told no | Decided no | -.28 | .137 | .254 | -.64 | .08 |
|  |  | No response | .12 | .131 | 1.000 | -.23 | .46 |
|  |  | Didn’t ask | -.24^*^ | .078 | .013 | -.45 | -.03 |
|  | Decided no | Told no | .28 | .137 | .254 | -.08 | .64 |
|  |  | No response | .39 | .168 | .112 | -.05 | .84 |
|  |  | Didn’t ask | .04 | .130 | 1.000 | -.31 | .38 |
|  | No response | Told no | -.12 | .131 | 1.000 | -.46 | .23 |
|  |  | Decided no | -.39 | .168 | .112 | -.84 | .05 |
|  |  | Didn’t ask | -.36^*^ | .125 | .025 | -.69 | -.03 |
|  | Didn’t ask | Told no | .24^*^ | .078 | .013 | .03 | .45 |
|  |  | Decided no | -.04 | .130 | 1.000 | -.38 | .31 |
|  |  | No response | .36^*^ | .125 | .025 | .03 | .69 |
| How did not being able to donate make you feel? - That breastfeeding wasn’t valued | Told no | Decided no | .95^*^ | .179 | <.001 | .47 | 1.42 |
|  |  | No response | -.78^*^ | .173 | <.001 | -1.24 | -.33 |
|  |  | Didn’t ask | -.23 | .103 | .155 | -.50 | .04 |
|  | Decided no | Told no | -.95^*^ | .179 | <.001 | -1.42 | -.47 |
|  |  | No response | -1.73^*^ | .220 | <.001 | -2.31 | -1.15 |
|  |  | Didn’t ask | -1.18^*^ | .171 | <.001 | -1.63 | -.73 |
|  | No response | Told no | .78^*^ | .173 | <.001 | .33 | 1.24 |
|  |  | Decided no | 1.73^*^ | .220 | <.001 | 1.15 | 2.31 |
|  |  | Didn’t ask | .55^*^ | .164 | .005 | .12 | .99 |
|  | Didn’t ask | Told no | .23 | .103 | .155 | -.04 | .50 |
|  |  | Decided no | 1.18^*^ | .171 | <.001 | .73 | 1.63 |
|  |  | No response | -.55^*^ | .164 | .005 | -.99 | -.12 |
| How did not being able to donate make you feel? - Disappointed | Told no | Decided no | .25 | .120 | .209 | -.06 | .57 |
|  |  | No response | -.10 | .115 | 1.000 | -.40 | .21 |
|  |  | Didn’t ask | .23^*^ | .069 | .006 | .05 | .41 |
|  | Decided no | Told no | -.25 | .120 | .209 | -.57 | .06 |
|  |  | No response | -.35 | .147 | .100 | -.74 | .04 |
|  |  | Didn’t ask | -.03 | .114 | 1.000 | -.33 | .28 |
|  | No response | Told no | .10 | .115 | 1.000 | -.21 | .40 |
|  |  | Decided no | .35 | .147 | .100 | -.04 | .74 |
|  |  | Didn’t ask | .33^*^ | .109 | .018 | .04 | .62 |
|  | Didn’t ask | Told no | -.23^*^ | .069 | .006 | -.41 | -.05 |
|  |  | Decided no | .03 | .114 | 1.000 | -.28 | .33 |
|  |  | No response | -.33^*^ | .109 | .018 | -.62 | -.04 |
| How did not being able to donate make you feel? - It felt unfair that I couldn’t donate but others could | Told no | Decided no | .83^*^ | .170 | <.001 | .38 | 1.28 |
|  |  | No response | -1.00^*^ | .163 | <.001 | -1.43 | -.57 |
|  |  | Didn’t ask | .04 | .097 | 1.000 | -.22 | .30 |
|  | Decided no | Told no | -.83^*^ | .170 | <.001 | -1.28 | -.38 |
|  |  | No response | -1.83^*^ | .208 | <.001 | -2.38 | -1.28 |
|  |  | Didn’t ask | -.79^*^ | .161 | <.001 | -1.22 | -.36 |
|  | No response | Told no | 1.00^*^ | .163 | <.001 | .57 | 1.43 |
|  |  | Decided no | 1.83^*^ | .208 | <.001 | 1.28 | 2.38 |
|  |  | Didn’t ask | 1.04^*^ | .155 | <.001 | .63 | 1.45 |
|  | Didn’t ask | Told no | -.04 | .097 | 1.000 | -.30 | .22 |
|  |  | Decided no | .79^*^ | .161 | <.001 | .36 | 1.22 |
|  |  | No response | -1.04^*^ | .155 | <.001 | -1.45 | -.63 |
| How did not being able to donate make you feel? - Guilty that I couldn’t donate | Told no | Decided no | .10 | .169 | 1.000 | -.35 | .54 |
|  |  | No response | -.63^*^ | .162 | <.001 | -1.06 | -.21 |
|  |  | Didn’t ask | -.05 | .097 | 1.000 | -.30 | .21 |
|  | Decided no | Told no | -.10 | .169 | 1.000 | -.54 | .35 |
|  |  | No response | -.73^*^ | .207 | .003 | -1.28 | -.18 |
|  |  | Didn’t ask | -.14 | .161 | 1.000 | -.57 | .28 |
|  | No response | Told no | .63^*^ | .162 | <.001 | .21 | 1.06 |
|  |  | Decided no | .73^*^ | .207 | .003 | .18 | 1.28 |
|  |  | Didn’t ask | .59^*^ | .154 | <.001 | .18 | .99 |
|  | Didn’t ask | Told no | .05 | .097 | 1.000 | -.21 | .30 |
|  |  | Decided no | .14 | .161 | 1.000 | -.28 | .57 |
|  |  | No response | -.59^*^ | .154 | <.001 | -.99 | -.18 |
| Based on observed means.  The error term is Mean Square(Error) = 1.300. | | | | | | | |
| *. The mean difference is significant at the .05 level. | | | | | | | |
